# Strain‐Mediated Anisotropic Growth of Metal–Organic Framework Belts

**DOI:** 10.1002/smtd.70745

**Published:** 2026-06-02

**Authors:** Valeriia Poliukhova, Hsien Cheng Huang, Vladimir V. Tsukruk

**Affiliations:** ^1^ School of Materials Science and Engineering Georgia Institute of Technology Atlanta Georgia USA

**Keywords:** anisotropic growth to belt morphology, Fe_3_O_4_ magnetic nanoparticles, metal–organic frameworks, phase stability via interfacial stabilization, pH‐controlled morphology, zeolitic imidazolate framework‐L

## Abstract

Metal–organic frameworks (MOFs), such as zeolitic imidazolate framework‐L (ZIF‐L), are organized porous materials that are sensitive to synthesis and processing conditions. Here, we report a one‐pot, purely aqueous route that couples pH‐controlled anisotropic growth of ZIF‐L platelets to unique belt morphologies and surface functionalization with citric‐acid‐capped Fe_3_O_4_ magnetic nanoparticles. This interfacial stabilization control of originally metastable layered MOF phases yields magnetically responsive nanomaterials without organic solvents, surfactants, or post‐synthetic processing. By adjusting the basicity of the 2‐methylimidazole environment, conventional ZIF‐L leaf platelets reproducibly evolve into high‐aspect‐ratio, elongated, belt‐like platelets with an aspect ratio of ∼10, while retaining the layered organization of the original ZIF‐L phase. Plane‐resolved x‐ray peak shifts (Δd/d ratio) show distinct, anisotropic lattice distortions, suggesting a stabilization mechanism in which interfacial coordination of carboxylate groups to under‐coordinated surface Zn^2+^ sites moderates layer relaxation and reconstruction pathways during synthesis and nanoparticle addition. This approach provides unique MOF belt morphology with enhanced phase stability and may be suggested as a scalable strategy for producing stable, anisotropic, magnetically responsive MOF‐based nanocomposites with non‐traditional shapes.

## Graphical abstract

An aqueous, one‐pot synthesis redirects layered ZIF‐L from conventional leaf morphology to high–aspect‐ratio belts (∼ 10) by pH‐controlled anisotropic growth, with magnetic nanoparticles decorating the platelet surfaces (MZIF‐L), improving phase stability during post‐synthetic handling. The morphological evolution is linked to compressive lattice distortion, consistent with the contraction of reflections in the lateral directions during anisotropic elongation.

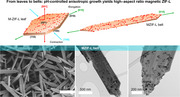

## 1 Introduction

Metal–organic frameworks (MOFs) are renowned for their exceptional combination of synthetic flexibility, various topologies, and tunable porosity, achieved through the unique modular coordination chemistry of ligands and metal nodes, which enable gas sensitivity and selectivity [1]. MOF structure, reactivity, and dynamic behavior are influenced by the variety of metal–ligand bonding, coordination, and interactions, changing behavior and properties [2, 3]. The foundational studies established the groundwork in the MOF field by discovering and characterizing the first crystalline permanently porous MOF‐5 by combining Zn_4_O nodes with 1,4‐benzenedicarboxylate (BDC) [4]. Subsequent studies expanded the field with MIL‐101(Cr), offering ultra‐large pores and record surface area [5], UiO‐66(Zr) delivering exceptional chemical and mechanical stability [6], HKUST‐1 (Cu‐BTC) showcasing open copper sites for strong gas adsorption [7], and MOF‐74 providing dense arrays of coordinatively unsaturated metal sites for highly selective CO_2_ capture [8]. These highlight how modular coordination chemistry enables both structural diversity and function, motivating continued efforts to precisely control MOF crystal chemistry and morphology, preserving its stability [9].

The concept of using imidazolate linkers to create MOFs was established to create zeolite‐like topologies, named zeolitic imidazolate frameworks (ZIFs) that mimic tetrahedral bonding with M–Im–M (metal‐imidazolate‐metal), where the bond angle is ∼145°, close to that of the Si–O–Si in zeolites [10, 11]. An extensive 3D ZIF portfolio was developed through high‐throughput synthesis, featuring various structures and functionalities, as well as high chemical and thermal robustness, adjustable pores and cages, which enable selectivity for CO_2_ capture and storage [12]. The most widely studied ZIF is 3D ZIF‐8 for gas separation, storage, catalysis, and more, with reported morphologies spanning rhombic dodecahedra and their truncated variants to perfect cubes, hollow and yolk–shell architectures, core–shell structures like Au@ZIF‐8 and Fe_3_O_4_@ZIF‐8, and even highly oriented ZIF‐8 thin membranes [13, 14, 15, 16, 17, 18]. Among the many morphological variants, a new 2D leaf‐like material, known as ZIF‐L, appeared: a layered polymorph of ZIF‐8 that crystallizes as thin nanosheets rather than 3D polyhedra [19].

2D layered architecture of ZIF‐L encompasses a cushion‐shaped cavity between layers, where two crystallographically distinct Zn(II) ions adopt regular [ZnN_4_] tetrahedral geometries and sheets are held together via hydrogen bonds involving “free” or monodentate 2‐methylimidazole molecules between the layers, yielding a leaf‐like morphology that differs from the fully 3D sodalite continuous cubic network of ZIF‐8 [19, 20]. This layered architecture offers a higher proportion of exposed active sites and shorter diffusion paths than ZIF‐8, making ZIF‐L more attractive for catalysis, sensing, and fast mass‐transport separations where surface accessibility is critical. For example, ZIF‐L@ZIF‐8 hybrid membranes showed three‐times higher hydrogen permeability and improved H_2_/CO_2_ selectivity due to ZIF‐L's layered structure combined with oriented ZIF‐8 growth [21]. The higher surface reactivity of ZIF‐L is related to uncoordinated or weakly coordinated 2‐methylimidazole, in which many surface atoms are close to the outer surface or edges, making surface sites more chemically accessible, and, thus, more reactive, for instance, toward organophosphorus molecule degradation and for acetylcholinesterase reactivation [22]. Despite extensive work on ZIF chemistry, reproducible aqueous morphology control of ZIF‐L while retaining the layered phase during post‐processing remains challenging.

Often described as a ZIF‐L to ZIF‐8 transformation, this process is better viewed as the intrinsic instability of ZIF‐L, which readily collapses or reconstructs into the thermodynamically favored ZIF‐8 by gradually losing its “free” ligand content when exposed to various solvents or washing conditions [23, 24, 25]. Prior reports show that this instability is accompanied by localized surface strain and lattice distortion (apparent strain), which may lower the barrier for reconstruction even in the absence of direct chemical or thermal degradation. Here, strain refers to apparent plane‐specific Δd/d derived from peak‐position shifts, which can capture anisotropic distortion from microstrain, stacking disorder, and guest redistribution; we use it as a mechanistic indicator rather than a direct measure of purely elastic stress.

The structural motif of ZIF‐L provides high surface accessibility, but also introduces a processing‐sensitive, metastable behavior that enables surface reconstruction under solvent, washing, external exposure, and drying perturbations [23, 24, 26]. Preserving the pure ZIF‐L phase while changing final morphology is difficult, as often the morphology change is tied to partial or full transformation to ZIF‐8, depending only on Zn: ligand ratio, solvent volume, and processing conditions [25]. The (001) surface of the ZIF‐L leaf has much lower surface energy than lateral facets like (100), meaning the crystal grows fast laterally in that direction, making wide flat leaves, while slower growth is observed in the other directions [20]. Overall, the MOF layered phase and 2D platelet morphology are often not retained through routine aqueous handling and drying, which complicates scale‐up and integration into a stable global organization [27]. It is well known that ZIF‐8 has a fully saturated, hydrophobic 3D network with few reactive Zn sites; conversely, direct aqueous surface‐shell functionalization of layered ZIF‐L remains underexplored.

In this work, we develop a novel one‐pot aqueous strategy that couples pH‐driven anisotropic growth of ZIF‐L leaves to form unique stable belt‐like morphologies. In this approach, we decorated MOF surfaces with modified Fe_3_O_4_ magnetic nanoparticles (MNPs), producing magnetically responsive ZIF‐L structures (MZIF‐L) and forming a non‐traditional belt morphology. The presence of MNP drastically improves retention of the 2D ZIF‐L phase during post‐synthetic handling and drying, preventing reconstruction to ZIF‐8. We suggest that the basic processing conditions and the presence of surrounding MNPs mitigate the buildup of anisotropic lattice strain, thereby promoting a more stable belt‐like anisotropic morphology that grows preferentially along the [010] direction of the leaf and is compressed in the lateral [100] direction, in contrast to the initial structure. ZIF‐L possesses exposed undercoordinated Zn^2+^ sites on its flat (100) face with a more hydrophilic external surface, giving Lewis‐acidic spots that readily bind the carboxylate groups of citric acid‐coated MNPs, in contrast to existing approaches [10, 16, 28]. The present one‐pot route addresses this by combining an aqueous pH morphology switch with nanoparticle‐based interfacial stabilization, enabling reproducible synthesis of ZIF‐L with unique anisotropic belt morphology.

## 2 Results and Discussion

### 2.1 Synthetic Strategy and Approaches

**FIGURE 1:**
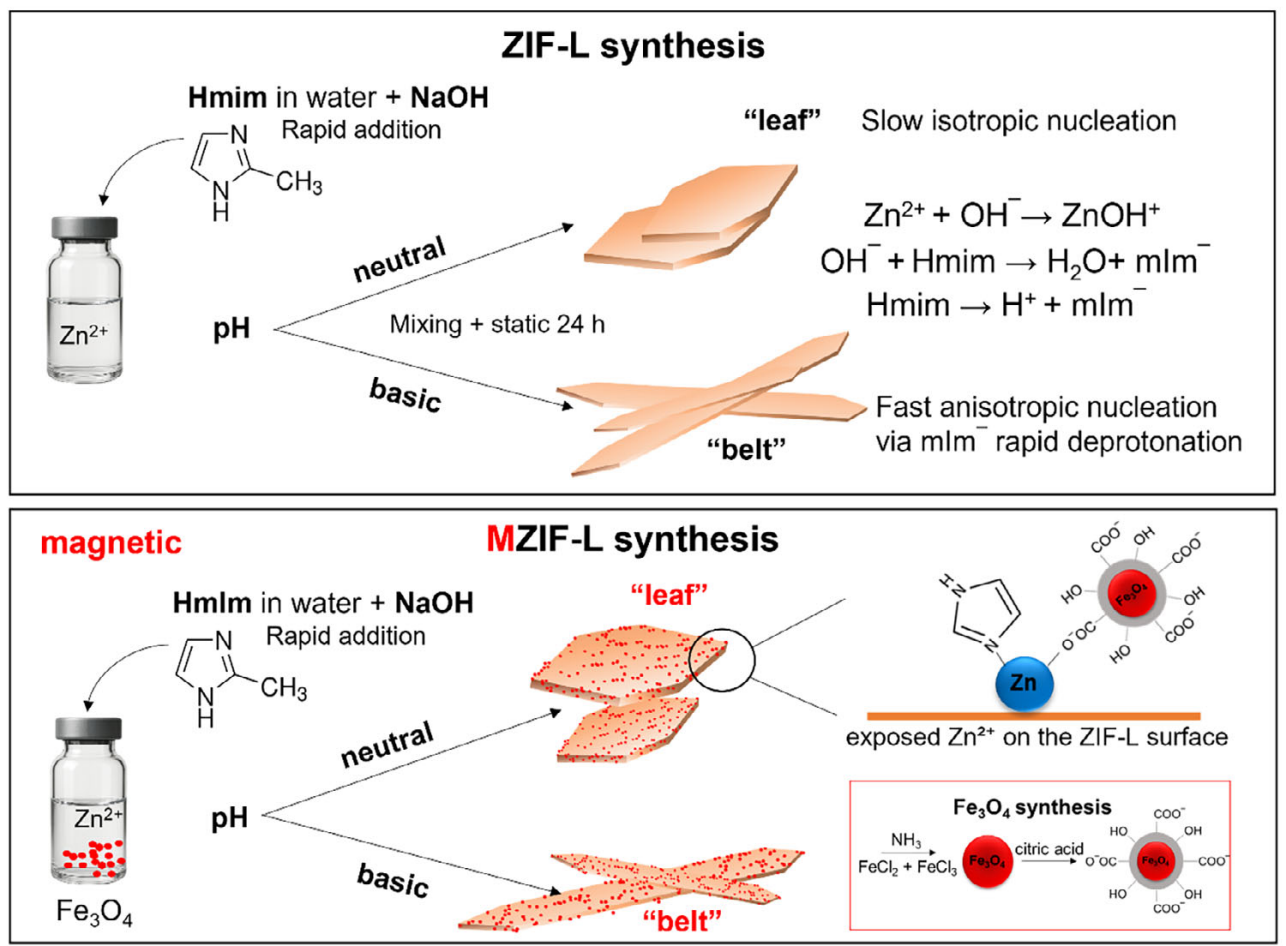
Illustrations of the developed synthetic procedures by modifying the pH of the aqueous reaction media, where natural conditions lead to a typical 2D “leaf” morphology, and basic conditions produce anisotropic “belt” morphology. The top panel shows ZIF‐L synthesis and nucleation mechanism, and the bottom panel shows the same synthesis conditions while using Fe_3_O_4_ magnetic nanoparticles, which deposit on the surfaces of ZIF‐L through exposed Zn^2+^ metal sites in situ during the ZIF‐L formation.

To control crystal growth in MOFs along certain lattice directions and achieve diverse morphologies, additives must be adsorbed on those facets during crystallization [29]. Tailoring the pH of the reaction media to a more basic pH is known to cause rapid linker deprotonation, inducing a faster nucleation rate and producing smaller crystals [29, 30]. A standard operating procedure for ZIF‐L synthesis can be tailored by refining critical parameters that influence the resulting morphology and particle sizes when water is used as the solvent, as explored in this study (Figure 1).

We propose that basicity modulates the balance between ligand deprotonation and metal species in solution, including the coordinating imidazolate form (mIm^−^) and hydrolyzed Zn–OH groups. Increased pH is expected to increase the fraction of deprotonated imidazole linkers and alter Zn^2+^ coordination, thus changing supersaturation, nucleation density, and facet‐dependent attachment rates, shifting growth from slow, near‐equilibrium toward fast, anisotropic growth. The excess of OH^−^, and Zn–OH species from NaOH (see Methods) can act as a coordination modulator that competes for Zn^2+^, altering attachment rates at specific crystal terminations, and can selectively cap high‐energy planes. We suggest that under these conditions, lateral widening of the ZIF‐L appears to be restricted, while elongation along the projected [010] direction is favored, resulting in the anisotropic belt‐like morphology with a high aspect ratio reaching 8–10, rather than a wide flat leaf platelet.

Importantly, this transition is achieved without changing the precursors or addition of surfactants in an aqueous environment, making the synthesis “green”, while the reaction yields a near‐quantitative yield of 85–100% with consistent reproducibility and opportunities for scalability. However, because ZIF‐L is a processing‐sensitive 2D layered polymorph that contains weakly bound ligand species between layers, this brings structural instability that depends on subtle changes in solution chemistry and post‐synthetic handling. To tackle this problem and introduce other advantages in the system, like magnetic recovery and response, we utilize citrate‐capped Fe_3_O_4_ magnetic nanoparticles in situ during the synthesis of ZIF‐L (named as MZIF‐L), leaving the rest of the procedure unchanged (Figure 1, bottom panel; see Methods for details) [19, 31].

A central rationale for including pre‐synthesized MNPs in the synthesis is to create stable interfaces on MOF surfaces, thereby protecting ZIF‐L from reconstruction into the more thermodynamically stable 3D ZIF‐8, known as a phase transformation [25]. As shown on the schematic, citric acid, which is typically used to prevent nanosized MNPs from aggregation, here binds strongly to the Fe_3_O_4_ surface through multidentate carboxylate interactions, leaving outward‐facing –COO^−^ groups [32]. During the ZIF‐L synthesis in the presence of MNPs, they approach the ZIF‐L surface that presents accessible Lewis‐acidic metal sites or defects, and form metal–carboxylate coordination bonds via chelation, which is stronger than just diffuse electrostatic attraction alone between the negative citrate shell and positive zinc metal sites. The adsorption of the functionalized nanoparticles onto the ZIF‐L surfaces is mediated by electrostatic attractions, while attachment occurs via Zn^2+^–COO^−^ bonding, accompanied by hydrogen bonding to surface groups and mediation by interfacial water. A key advantage of the obtained MZIF‐L macrostructures is magnetic separation, enabling particle recovery, where Fe_3_O_4_ on surfaces decreases surface–surface face‐to‐face contact area, reducing restacking‐driven reconstruction.

### 2.2 Synthetic Steps and Morphology Evolution

Practical synthetic steps for MOF synthesis and particle size control include careful selection of solvent, precursor type and ratio, coordination modulation, deprotonation kinetics, and post‐processing; however, most MOFs still possess only a single morphology since they are derived from a single synthetic procedure [29]. For ZIF‐8, a widely used method is the rapid room‐temperature route in alcohols (often methanol), in which Zn(II) salt and excess 2‐methylimidazole are prepared as separate solutions, then mixed under vigorous stirring [10, 12, 33, 34]. A common aqueous strategy utilizes NH_4_OH, NaOH, or other bases to accelerate *Hmim* deprotonation, enabling fast ZIF‐8 formation and control over particle size and truncated‐cubic morphology [35]. ZIF‐L is most often prepared by aqueous, room‐temperature reactions of Zn(II) salts with 2‐methylimidazole that yield the characteristic 2D leaf‐like crystallites, with growth influenced by concentration, aging time, and solvent modulation with alcohols, along with polymer or surfactant additives [19, 36]. Importantly, multiple studies treat ZIF‐L as a processing‐sensitive intermediate on the route to ZIF‐8, where solvent choice in synthetic or post‐processing steps can accelerate phase evolution [23, 24, 25].

Herein, we prepared ZIF‐L_leaf microparticles in water as the solvent, following the standard route, by tailoring the precursor ratio and total volume, and using the general synthesis conditions of the previously reported procedure [19]. To obtain ZIF‐L_belt morphology, the pH of the 2‐methylimidazole was raised by adding concentrated NaOH before mixing with Zn metal solution, where the final pH was 9 (see Methods). The ZIF‐L_leaf and ZIF‐L_belt particles obtained without MNPs are shown in the scanning electron microscopy (SEM) images in Figure S1a,b. Leaf has a general 2D wide platelet morphology with average dimensions: width of 2.5 ± 0.44 µm, length of 6.84 ± 1.32 µm, and a modest aspect ratio of 2.7 ± 0.23 (Figure S1c). In contrast, the belt morphology exhibits a greatly elongated platelet shape with a significantly higher aspect ratio. Indeed, the high aspect ratio of 8.23 ± 1.32 µm results from a decrease in the average platelet width to 0.96 ± 0.13 µm, along with an increase in length to 7.82 ± 1.1 µm (Figure S1c).

**FIGURE 2:**
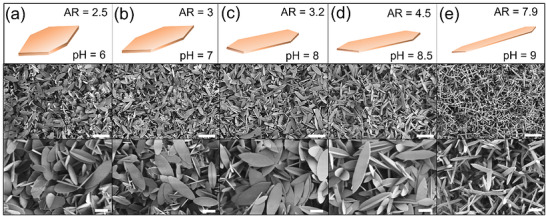
SEM images showing transition from MZIF‐L_leaf to MZIF‐L_belt morphologies (left to right) produced by changing the pH of the aqueous medium during MZIF‐L synthesis with schematic representation of each particle dimensions with their respective aspect ratio (AR) and solution pH, with respective images of the obtained samples below with a scale bar of 20 µm in the mid panel and scalebar of 5 µm in the bottom panel, respectively. Typical leaf morphology is synthesized without the addition of NaOH, at pH 6, e.g., MZIF‐L_leaf (a), and increasing pH from 7 to 9 (b to e), respectively, where (e) represents belt morphology or MZIF‐L_belt.

Next, Fe_3_O_4_ magnetic nanoparticles (magnetite, space group Fd3m, a ≈ 8.37 Å) with an average size of 6.5±1.2 nm were pre‐synthesized via the co‐precipitation method described previously (see Methods and Figure S2) [31]. The resultant MNPs had peak positions at 30.1° (220), 35.5° (311), 43.1° (400), 53.5° (422), 57.0° (511), 62.6° (440), attributed to standard magnetite JCPDS 19–0629 [37]. These MNPs were then used to decorate the surfaces of ZIF‐L in a desired concentration and pre‐mixed with Zn salt before the addition of the linker to obtain MZIF‐L (see Methods). To investigate how the particles’ shape transforms from leaf to belt, evaluate particle sizes, and calculate their aspect ratios, the MZIF‐L aqueous medium was tailored by gradually adjusting the pH from 6 to 9 using NaOH during the addition of imidazole linker to the metal aqueous solution in the presence of MNPs (Figure 2).

To quantify this analysis, we consider each morphology using the calculated in‐plane aspect ratio (AR, length along [010] direction divided by width). Most studies on ZIF‐L report only their lateral dimensions with a low aspect ratio of ∼3–6 µm long and 1.5–2.5 µm wide [24, 38]. Evidently from our investigation, when no alkali is used, and the pH of the aqueous medium is 6, typical MZIF‐L_leaf shapes possess larger than reported dimensions: an average width of 4.3 ± 0.6 µm, length of 10.8 ± 2.2 µm, and modest aspect ratio of 2.5 ± 0.4 (Figure 2a).

**FIGURE 3:**
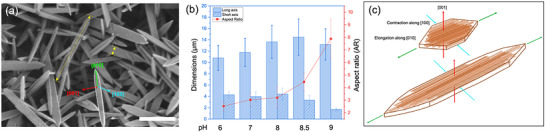
SEM micrograph of MZIF‐L_belt showing representative belt morphology, particle length/width marked in yellow, and projected [001], [010], and [100] directions associated with leaf morphology evolution into belt, scale bar 5 µm (a). Average long‐axis length, short‐axis width, and calculated aspect ratio of MZIF‐L particles synthesized at different pH values, where error bars represent standard deviation (b). Schematic illustration of the proposed anisotropic growth model, where [001] corresponds to the particle thickness direction, lateral growth is restricted along [100], and elongation proceeds preferentially along the [010] direction (c).

The larger leaf dimensions are attributed to the modified synthesis conditions, in which, instead of stirring or agitation, an ice sonication bath was used to mix the precursors (see Methods). When the pH increased to 7, 8, 8.5, and 9, the mean particle lengths increased progressively up to pH 8.5, while the mean width kept gradually decreasing up to pH 9 (Figure 2b–e). Finally, at the highest pH, the completely formed belt particles are shown at higher magnification (Figure 3a) and exhibit an average width of 1.7 ± 0.25 µm, length of 13.2 ± 2.8 µm, and AR of 7.9 ± 1.5 (Figure 3b; see also Figure S3 for AR distribution and particle counts). These values are substantially higher than typical literature values for conventional ZIF‐L platelets [19, 24, 37].

**FIGURE 4:**
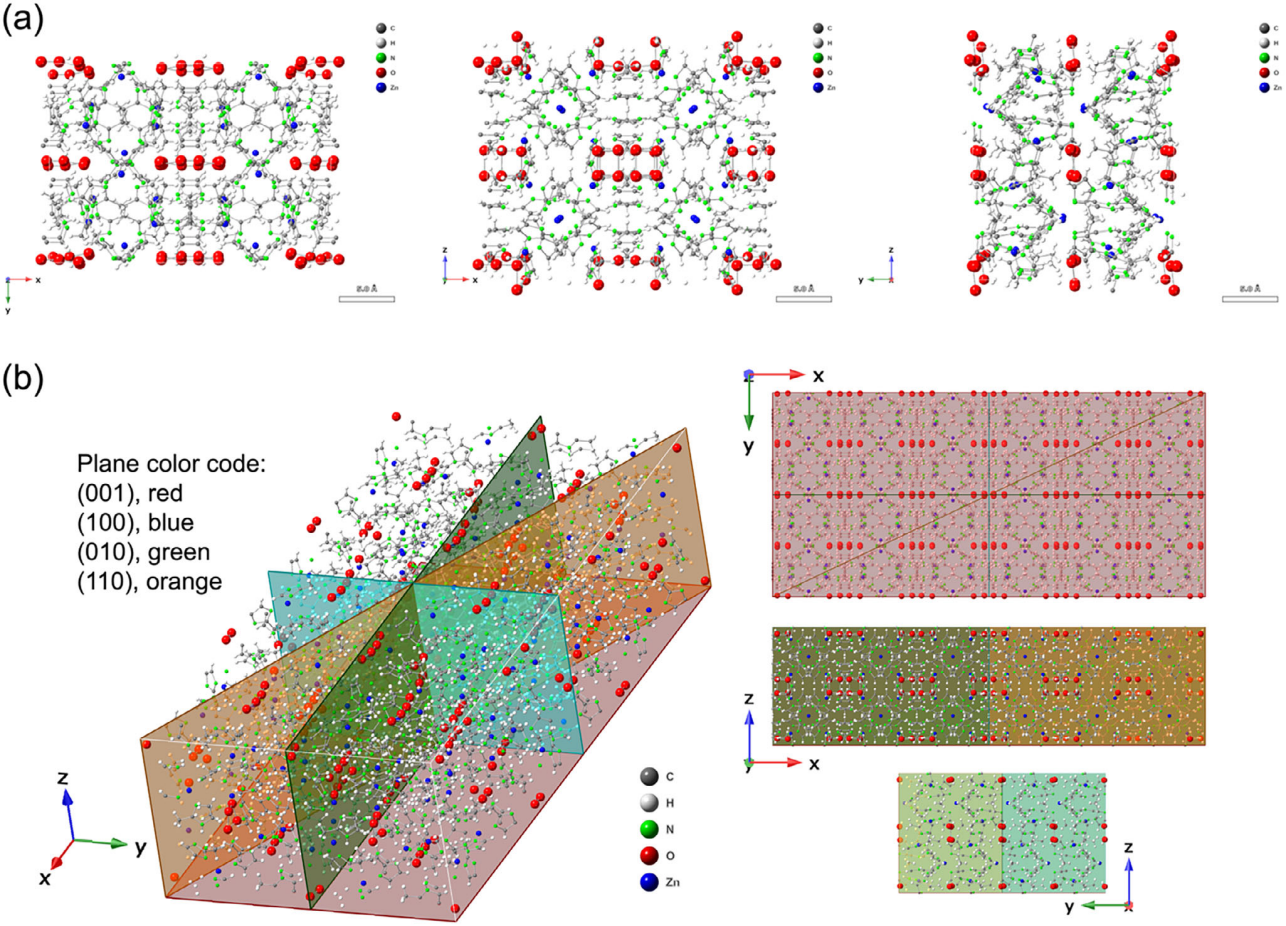
The ZIF‐L crystal‐structure model and crystallographic plane assignment used to rationalize the belt‐like morphology. ZIF‐L crystal structure rendered in CrystalMaker from a reported ZIF‐L crystallographic information file (CIF) [19, 24], shown as orthographic projections along the principal crystallographic directions. The unit‐cell orientation is retained to show the layered framework geometry. Atom colors are Zn, purple; N, blue; C, gray; O, red; and H, white, (a). Selected low‐index crystallographic planes, (001), (100), (010), and (110), are rendered within the same ZIF‐L structural model using their Miller indices. The projected views on the right show the orientation of each highlighted plane relative to the lattice. These crystallographic projections are used to support the projected [001], [100], and [010] directions shown in Figure 3, where [001] corresponds to the particle thickness direction, [100] to the restricted lateral‐growth direction, and [010] to the preferential elongation direction.

The belt morphology observed correlates with the ZIF‐L crystal structure, referencing the directions shown in Figure 3a and based on the ZIF‐L model created in CrystalMaker from the CIF data in prior ZIF‐L research (Figure 4) [19, 24]. The projections serve as a foundation for connecting the observed growth directions in the belt morphology and explaining the leaf‐to‐belt transformation. Viewing the ZIF‐L structure along key crystallographic axes reveals the layered framework and the relevant planes (001), (100), (010), and (110), marked in red, blue, green, and orange, respectively (Figure 4). This model helps visualize how the leaf transforms into a belt, with [001] indicating thickness, [100] lateral growth, and [010] elongation [19, 24]. According to this model, the belt forms prompted by the basicity change in water, and can be seen as a directional growth imbalance—favored along [010] while suppressing lateral widening along [100].

Thus, we suggest an anisotropic growth mechanism in which the belt extends along [010], the thickness increases along [001], and lateral growth along [100] is limited. These designations are based on a projected crystallographic model in Figure 4 to support the upcoming X‐ray diffraction (XRD) discussion, not as direct crystallographic indexing from SEM.

Overall, pH‐dependent belt formation is best described as a proposed kinetically controlled anisotropic‐growth pathway, consistent with increasing basicity accelerating deprotonation of *Hmim* to the coordinating imidazolate form (mIm^−^) and modifying Zn^2+^ hydrolysis and speciation mechanism [29, 30], discussed earlier, thereby changing both nucleation density and the relative rates of lateral vs. longitudinal growth in the layered ZIF‐L. In a layered framework, such as ZIF‐L, this anisotropic growth can be rationalized by a coupled kinetic and surface‐energy effect, in which the supply rate of building units and the stabilization of specific facets in the presence of OH^−^ and deprotonated ligand shift the dominant growth direction.

Accordingly, increasing basicity does not merely accelerate crystallization; it shifts the growth regime, favoring elongation and yielding belts with high aspect ratios rather than the well‐studied leaf platelets. At pH values above 9, the products instead evolve toward more isotropic, near‐spherical particles (see Figure S4), which is consistent with a transition to a high‐supersaturation, high‐nucleation regime and competing ZnOH^+^ precipitation pathways that reduce facet‐selective growth. Thus, the results discussed indicate that there is an optimal pH window in which ZIF‐L anisotropy emerges.

Additionally, changing the total reaction volume reveals a clear shift in growth regime. Under the standard conditions used for the leaf morphology, the total water volume was 20 mL (Figure 2a). Decreasing the volume yields smaller, thicker platelets, whereas increasing the volume produces larger, flatter, sheet‐like particles with more rectangular‐shaped edges (Figure S5). This behavior is consistent with dilution lowering effective supersaturation and collision frequency, thereby suppressing nucleation, allowing fewer nuclei to grow larger based on diffusion‐mediated modulation in layered MOFs [39].

### 2.3 MOF Decoration with Magnetic Nanoparticles

**FIGURE 5:**
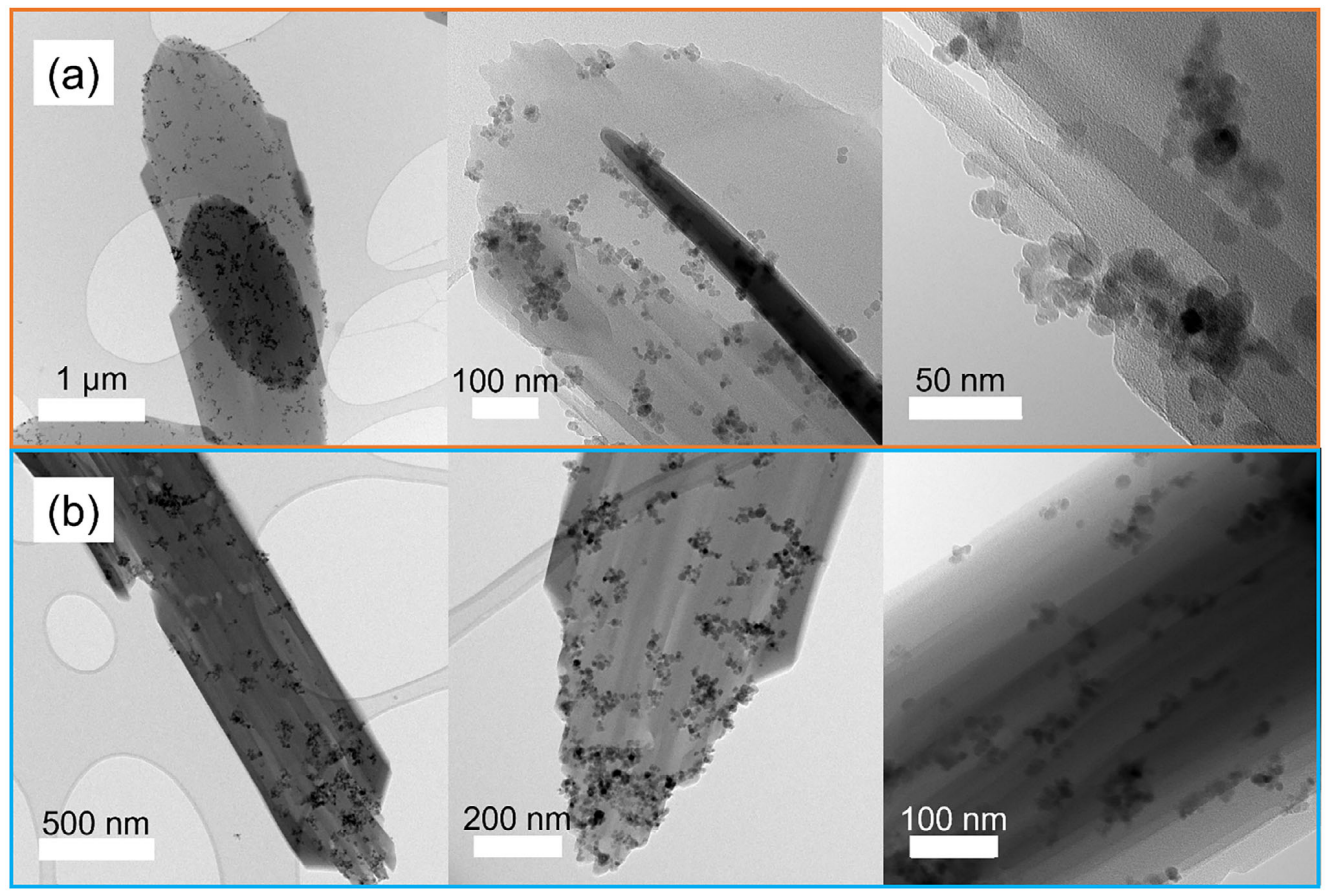
TEM micrographs of the MZIF‐L_leaf (a) and MZIF‐L_belt (b) layered morphologies at various magnifications, showing the surface attachment of Fe_3_O_4_ magnetic nanoparticles.

Since the MNPs used in situ during the synthesis of MZIF‐L are too small to be visible in SEM (Figure S2), we conducted transmission electron microscopy (TEM) to visualize them on the ZIF‐L surfaces. The defining compositional feature of MZIF‐L is that Fe_3_O_4_ particles are not simply mixed or co‐precipitated as a separate phase from the ZIF‐L, but are attached to the ZIF‐L surface through citrate anchoring. Direct evidence of the MNPs homogeneously distributed on the surfaces of MZIF‐L_leaf and MZIF‐L_belt platelets can be seen in selected TEM in Figure 5, and their response to the magnet is shown in Figure S6. Supplementary TEM images of the MZIF‐L_belt sample captured in different locations with low and high magnifications are provided in Figure S7, as evidence of MNPs homogeneously distributed on the surfaces and layers of ZIF‐L on a larger scale. Additionally, from TEM images, we estimated the leaf morphology thickness to be 74 ± 52 nm, which increased to 273 ± 68 nm for the belt.

The attachment of Fe_3_O_4_ MNPs to the ZIF‐L particles is supported by their magnetic separation behavior, as shown in the photographs, from the aqueous dispersions of MZIF‐L_leaf and MZIF‐L_belt, which migrate toward the vial wall when a conventional permanent magnet is placed adjacent to the container (Figure S6). This indicates that the composite particles are magnetically responsive due to the presence of MNPs attached to the ZIF‐L surface. The MZIF‐L suspensions also exhibit a uniform light orange‐brown coloration, originating from the intrinsic optical properties and electronic structure of Fe^2^^+^/Fe^3^^+^ in MNPs [40]. Thus, when Fe_3_O_4_ nanoparticles are attached to the ZIF‐L surface, the composite particles take on the visual signature of the iron oxide, where the orange‐brown coloration of the MNPs dominates the overall appearance of the otherwise white MOF host. Thus, this color change and the observed magnetic response are direct visual indicators of successful surface functionalization with Fe_3_O_4_ nanoparticles, along with the homogeneous distribution of Fe_3_O_4_ nanoparticles on the ZIF‐L surfaces (Figure 5; Figures S6 and S7).

These results are consistent with interfacial coordination of citrate carboxylate groups from MNPs to undercoordinated surface Zn^2+^ sites on ZIF‐L facets and edges, an energetically plausible interaction that is consistent with the microscopy evidence. Layered ZIF‐L exhibits more accessible coordination motifs and can be more hydrophilic, thereby enabling the adsorption or anchoring of Fe_3_O_4_ NPs on ZIF‐L surfaces.

Fourier transform infrared (FTIR) spectra were also collected as complementary characterization of chemical composition (Figure S8). These spectra remain similar for ZIF‐L and MZIF‐L samples, with only subtle changes in the imidazole ring bonds and C═N region, where bonding of Fe_3_O_4_ to imidazolate nitrogen can perturb electron density in the ring, thus suggesting preferential bonding. As a proof of concept, the MZIF‐L_leaf particles were synthesized with pre‐made MNPs, without the addition of citric acid to their surfaces, resulting in significant aggregation, as shown in the SEM image and a photograph of the particles after centrifugation, which shows a distribution of orange‐brown shades, indicating significant agglomeration and mixed phase appearance (Figure S9).

It is important to distinguish the roles of pH and the addition of Fe_3_O_4_ MNPs. The leaf‐to‐belt morphological transition is primarily governed by the pH‐controlled ZIF‐L growth regime, as uncoated ZIF‐L_belt particles are obtained under the same basic conditions without the presence of MNPs (Figure S1). In contrast, citrate‐capped Fe_3_O_4_ nanoparticles mainly provide magnetic response and interfacial stabilization after formation of the anisotropic ZIF‐L morphology. Thus, MNPs are not considered the origin of belt formation, but rather a surface‐bound stabilizing nanoparticulate component that helps preserve the original ZIF‐L phase during post‐synthetic handling and drying.

### 2.4 MOF Phase Stability and Reconstruction From Post‐Processing

A major practical outcome is that the apparent crystalline phase of uncoated ZIF‐L powders is highly sensitive to post‐synthetic drying history, and that this sensitivity is strongly mitigated by Fe_3_O_4_ MNP surface coating. Specifically, when uncoated ZIF‐L_leaf and ZIF‐L_belt powders are dried slowly under mild conditions (40°C, 24 h), their diffraction patterns convert to a ZIF‐8–type fingerprint (Figure S10a) even though prior to XRD measurement, both morphologies are visibly 2D leaf and belt shapes on the SEM (Figure S1), instead of the prevalent ZIF‐8 3D rhombic dodecahedron. During reported solvent washing‐driven reconstruction from ZIF‐L to ZIF‐8, leaf‐shaped ZIF‐L disappears and is replaced by smaller ZIF‐8 nanoparticles [24]. In our case, after the XRD analysis, SEM shows that ZIF‐L particles appear mechanically collapsed or squashed (Figure S10b).

**FIGURE 6:**
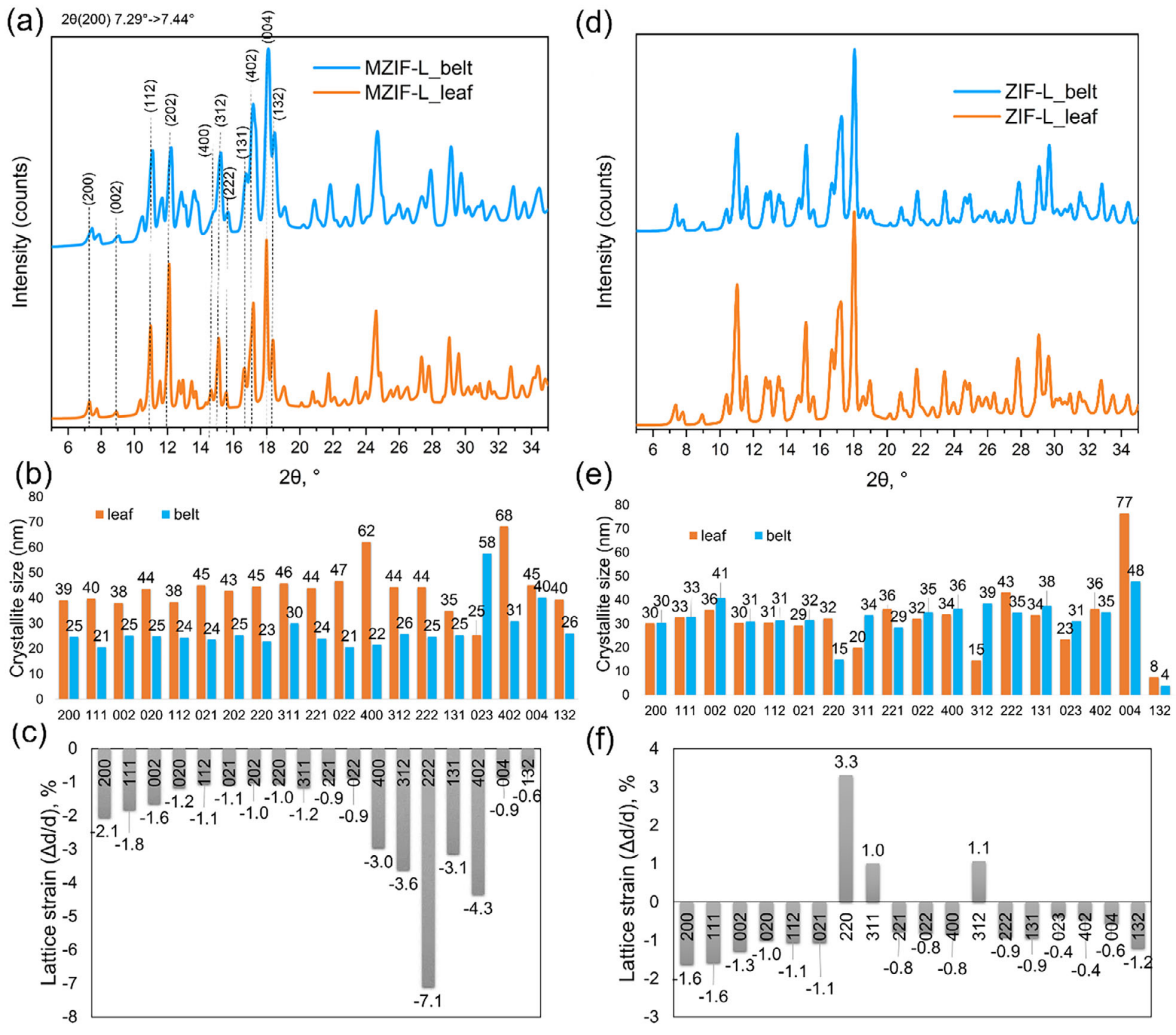
XRD data comparison of “belt” and “leaf” morphologies for (a–c) MZIF‐L (Fe_3_O_4_ coated) and (d–f) uncoated ZIF‐L. (a) Diffraction patterns (Cu Kα) with indexed ZIF‐L reflections, where MZIL‐L_belt shows systematic peak shifts to higher 2θ (e.g., (200) 7.29° → 7.44°); (b) Reflection‐resolved coherent domain sizes for MZIF_L estimated from peak broadening (Scherrer); (c) Plane‐resolved apparent lattice distortion for MZIF‐L calculated as Δd/d = (d_belt_−d_leaf_)/d_leaf_ × 100%, where negative values indicate contraction of the (hkl) spacing in belts relative to leaves; (d–f) the same data analysis as in (a–c) for ZIF‐L_belt and ZIF‐L_leaf. Reference ZIF‐L structural assignment is consistent with published data [19, 24].

This reorganization is consistent with significant dehydration and handling‐induced densification during sample mounting and measurement rather than a required macroscopic shape change. In contrast, when those same uncoated samples are dried more rapidly (at 60°C overnight), they retain the ZIF‐L diffraction pattern (Figure 6d). The drying dependence provides a direct connection between phase reconstruction and the plane‐resolved lattice distortion discussed below. ZIF‐L contains weakly bound or monodentate 2‐methylimidazole species and interlayer water. Thus, dehydration changes not only the solvent content but also the interlayer environment that supports the layered framework. Slow drying likely provides sufficient time for layer relaxation, guest redistribution, and local reconstructive rearrangement, allowing metastable ZIF‐L to evolve into the thermodynamically favored ZIF‐8. Faster drying, by contrast, can kinetically retain the layered ZIF‐L structure before extensive reconstruction occurs. This behavior aligns with prior reports that ZIF‐L can act as a kinetic or intermediate polymorph that transforms to the thermodynamically favored ZIF‐8 topology depending on processing conditions and transformation kinetics [23, 24, 25].

Thermogravimetric analysis (TGA) was further added to compare the thermal stability of ZIF‐L_leaf, ZIF‐L_belt, MZIF‐L_leaf, and MZIF‐L_belt samples (Figure S11). All samples show multistep mass loss, with lower‐temperature loss assigned to adsorbed or interlayer water and higher‐temperature loss associated with weakly bound or free ligand species and thermally‐induced framework degradation [24]. The transformation from ZIF‐L to ZIF‐8 is primarily a topological reconstruction of the Zn–imidazolate framework, and is not expected to produce a unique, isolated mass‐loss event in TGA; thus is used here as complementary thermal‐stability evidence. Finally, TGA data confirm the overall presence of 35–40 weight% (or 7–10% of total volume considering different densities) of magnetic nanoparticles in composite magnetic MOF materials (Figure S11).

The results for slow‐dried materials (Figure S10a) match the characteristic low‐angle ZIF‐8 reflections, with the strongest peaks at 2θ ≈ 7.35°, 10.41°, 12.76°, 14.74°, 16.49°, and 18.08° (ICDD PDF 02‐002‐8846), which belong to the sodalite (SOD) ZIF‐8 structure (space group I‐43m) [10, 11, 12]. In comparison, the ZIF‐L has an orthorlhombic layered phase (Figure 6a,d), contains distinct reflections at 2θ ≈ 7.32° (200), 7.76° (111), 8.95° (002), 10.36° (020), 10.98° (112), 11.58° (202), and 12.70° (220), along with additional ZIF‐L characteristic peaks at other positions at 13.47°, 13.71°, 15.12°, 17.00°, and 18.00° [19, 24].

Thus, the experimental switch between ZIF‐L and ZIF‐8 patterns with drying conditions is not a subtle shift but corresponds to the emergence of the ZIF‐8 peak set and suppression of ZIF‐L‐specific layered reflections and is not governed only by precursor ratios or solvent chemistry during synthesis, but can be triggered post‐synthetically by the dehydration pathway. This is consistent with mechanistic studies describing ZIF‐L as a transformation intermediate that often involves dissolution–reprecipitation or solvent‐mediated reconstruction, associated with monodentate and free *Hmim* between layers, which plausibly increases sensitivity to structural rearrangement during drying [24].

### 2.5 Stabilizing Role of Magnetic Nanoparticles on the MOF Phase

Within this framework, the stabilizing role of MNP on ZIF‐L becomes apparent, as MZIF‐L retains ZIF‐L diffraction fingerprint under drying conditions (Figure 6a), while uncoated ZIF‐L can be reconstructed during powder dehydration and handling (Figure S10a). Mechanistically, a surface‐bound nanoparticle shell can plausibly impede reconstruction by interacting with Lewis‐acidic surface metal sites and modify interfacial chemistry; the nanoparticle interlayer can reduce mass transport and suppress dissolution–reprecipitation cycles during drying that enable reconstruction.

Therefore, the combined SEM and XRD data demonstrate that particle morphology is not a definitive phase identifier in this system, meaning the transformation is a structural rather than morphological one. Furthermore, from the X‐ray data, we present a plane‐resolved microstructural comparison using Δd/d analysis of XRD peak positions to compare lattice distortions between MZIF‐L_leaf, MZIF‐L_belt, and their uncoated counterparts (Figure 6). This separates phase identification from microstructure (line broadening related to coherent domain size), and apparent lattice distortion (Δd/d changes derived from peak shifts). Because 2D crystallites can exhibit strong preferred orientation, relative peak intensities are not treated as a primary phase metric here; instead, we focus on peak positions and systematic shifts.

Both MZIF‐L_leaf and MZIF‐L_belt retain the ZIF‐L diffraction, consistent with the earlier conclusion that MNP coating stabilizes ZIF‐L against processing‐driven reconstruction (Figure 6a). The most direct evidence of a morphology‐dependent lattice response is that MZIF‐L_belt peaks are systematically shifted to higher 2θ relative to MZIF‐L_leaf across the majority of tracked reflections. This is readily seen at low angle for the (200) reflection, which shifts from 2θ = 7.29° (d = 12.1 Å) in leaf to 7.44° (d = 11.86 Å) in belt, corresponding to Δd/d = −2.1% (Figure 6a,c). The same trend persists through mid‐angle reflections, including (400) (14.32° → 14.75°; Δd/d = −3%) and becomes especially pronounced for several planes such as (222) (15.54° → 16.74°; Δd/d = −7.1%) and (402) (17.2° → 18°; Δd/d = −4.3%) (Figure 6c).

It is worth noting that not every plane contracts equally, and the magnitude of contraction is strongly plane‐dependent. However, the dominant sign is strongly negative (structural contraction in belts relative to leaves), indicating a single dominant relaxation mode rather than competing expansion and contraction pathways. In this view, the apparent Δd/d values derived from XRD peak shifts reflect anisotropic lattice response associated with different stress development due to drying, guest loss, stacking disorder, and constrained relaxation rather than purely elastic strain caused by a single cause.

This contraction‐dominated response is mechanistically plausible for ZIF‐L because it is a layered framework stabilized by interlayer interactions and free ligand content, in which changes in hydration, guest distribution, or local bonding during drying can alter plane spacings and stacking coherence without requiring macroscopic changes. The coherent domain sizes were estimated from peak broadening [41, 42]. (Figure 6b) show that MZIF‐L_belt has generally wider peaks with smaller domains than MZIF‐L_leaf across many reflections (e.g., from (200): leaf = 39 nm vs. belt = 25 nm; from (400): leaf = 62 nm vs. belt = 22 nm; from (222): leaf = 44 nm vs. belt = 25 nm), suggesting domain refinement during leaf to belt transformation in basic conditions. Such diffraction peak broadening can arise from a combination of finite coherent domain size and microstrain defect broadening, and the reflection‐to‐reflection variability is consistent with anisotropic disorder and stacking contributions typical of non‐cubic, layered materials [42, 43, 44].

For the uncoated samples, the belt and leaf patterns can retain the ZIF‐L fingerprint under the faster‐drying condition identified earlier (60°C overnight), but the plane‐resolved distortion map differs from MZIF‐L (Figure 6d–f). While several low‐angle reflections still show modest contraction in belts relative to leaves, (e.g., (200) 7.27° → 7.39°; Δd/d = −1.6%, (111) 7.7° → 7.83°; Δd/d = −1.6%, and (002) 8.9° → 9.02°; Δd/d = −1.3%), mixed‐sign behavior at higher angles is observed (Figure 6f). Most notably, the (220) reflection shifts to a lower 2θ for the belt sample (12.65° → 12.24°), yielding an expansive Δd/d = + 3.3%, and additional positive distortions appear for (311) (+1%) and (312) (+1%). This mixed‐signature pattern indicates that uncoated belts do not undergo a uniform contraction‐like response, and, instead, the lattice response is heterogeneous, which can indicate competing processes such as non‐uniform water loss, stacking disorder evolution, or defect gradients [45, 46, 47, 48].

In Figure 6e, the coherent domain sizes for uncoated samples are also more reflection‐dependent, where some planes broaden more in leaf and others in belt, reinforcing the conclusion that uncoated ZIF‐L experiences a less constrained microstructural evolution during processing. Thus, we can summarize that uncoated ZIF‐L exhibits heterogeneous, competing relaxation modes (Figure 6f) that, given sufficient time under mild drying, can progress toward reconstructive transformation (ZIF‐8‐like in Figure S10). This behavior is aligned with recent studies that treat ZIF‐L as a kinetically accessible layered intermediate phase whose conversion to ZIF‐8 is strongly dependent on processing and transformation kinetics [23, 24, 25, 26]. The obtained results in this study indicate that MNP coating does not merely preserve the external morphology of ZIF‐L, but correlates to robust phase retention, and a more coherent, contraction‐dominated lattice distortion pattern (Figure 6c). The presence of an MNP shell in MZIF‐L enables interfacial interactions that can limit surface rearrangement and mitigate the various growth pathways that lead to shape reconstruction, thus facilitating MOF phase transformation and retention under the given processing conditions.

## 3 Conclusions

The results of this study support the proposed mechanism of MOF shape control, in which pH alters 2‐methylimidazole deprotonation and Zn–imidazolate speciation, thereby redirecting ZIF‐L crystallization from the widely reported leaf‐like 2D platelets toward a unique high–aspect‐ratio belt morphology via anisotropic growth under basic conditions (Figure 1). In the reported studies, aqueous syntheses most commonly yield leaf or nanosheet morphologies, whereas other variants have typically been achieved using structure‐directing additives, such as PVP in ethanol [36]. In contrast, the one‐pot aqueous approach suggested here produces belt‐shaped layered ZIF‐L while simultaneously enabling surface functionalization with citrate‐capped Fe_3_O_4_ MNPs, providing a stabilizing interfacial layer that changes post‐synthetic handling.

The crystallographic‐direction assignment suggests that in basic conditions, leaf has preferential elongation along [010], restricted lateral growth along [100], and thickness increase along [001], which evolves into a belt structure. In parallel, introduced citrate‐capped Fe_3_O_4_ MNPs provide a magnetic response and interfacial stabilization into the MZIF‐L composite, improving retention of the ZIF‐L crystallographic phase during post‐synthetic handling and drying, preventing the mechanical collapse into ZIF‐8. Importantly, the results suggest that MZIF‐L is more robust against processing‐driven reconstruction as verified by XRD phase retention, due to a distinct, more uniform pattern of plane‐resolved Δd/d compared with uncoated samples.

Our analysis suggests that the higher‐energy facets are subject to compressive lattice distortion as ZIF‐L grows, consistent with contraction of reflections associated with lateral directions during anisotropic elongation. Thus, we propose that modifying growth conditions that affect surface energy, surface relaxation, and facet exposure could be a way to alter and preserve morphology by favoring or suppressing specific facets via pH, solvent, ligand concentration, or temperature. More broadly, the strategy developed in this study provides a transferable framework for controlling morphology and post‐processing stability in generally metastable 2D MOFs, where surface relaxation and reconstruction pathways frequently limit practical use.

Practically, the resulting belt‐shaped, high‐aspect‐ratio 2D ZIF‐L is more attractive for applications where orientation and interfacial contact area matter, especially in membranes and mixed‐matrix membranes, where aligned 2D MOF fillers can provide fast transport pathways and improved performance [44, 49, 50]. ZIF‐L is also actively explored in catalysis and water‐treatment adsorption, where the added magnetic recoverability is valuable for rapid solid–liquid separation and reuse in magnetically‐enriched MOF composites [51, 52, 53].

It is important to note that by decorating MOF surfaces with Fe_3_O_4_ nanoparticles and guiding strain‐assisted anisotropic growth, simultaneous phase stabilization, as well as shape and morphology control can be achieved in a single‐step aqueous synthetic procedure. This combination of shape preservation, phase stability, and final induced magnetic functionality opens new possibilities for consideration of magnetic high‐aspect ratio 2D MOF belt‐like materials in separations, catalysis, tailored membranes, and responsive nanocomposites well beyond current examples of conventional magnetically‐decorated MOFs [16, 51, 52, 53, 54].

## 4 Methods

### 4.1 Materials Components

All chemicals used were purchased from Sigma–Aldrich.

### 4.2 Magnetic Nanoparticles

Fe_3_O_4_ MNPs were prepared via the co‐precipitation method described previously [31]. Briefly, FeCl_2_·4H_2_O (1 g) and FeCl_3_·6H_2_O (2.7 g) precursors were mixed in 50 mL deionized (DI) water with vigorous stirring under nitrogen gas for 30 min, followed by a subsequent addition of 6 mL of NH_3_·H_2_O and stirred for an additional 30 min. Then, an aqueous solution of citric acid (1.5 g in 2 mL DI water) was added to the solution for stabilization, and the mixture was left continuously stirring for 2 h. Then Fe_3_O_4_ nanoparticles were collected after washing with DI water 4–5 times until the pH was neutral, and separated with the external magnetic field to remove the impurities and re‐dispersed in DI water at 0.3 wt.%. Ultrasound treatment by tip sonicator (40% amplitude) was applied for 10 min before further use of MNPs. The MNPs were measured with TEM and X‐ray diffraction to confirm their spherical morphology and phase.

### 4.3 Synthesis of MZIF‐L Leaves

Citrate‐capped Fe_3_O_4_ magnetic nanoparticles (MNPs) were first diluted in DI water to a concentration of 1 mg/mL. Zinc nitrate hexahydrate (Zn(NO_3_)_2_·6H_2_O) 0.5 mol/g stock solution (a) was prepared by adding 1.485 g of powder into 10 mL DI water. 2‐methylimidazole (2‐MeIm) 2 mol/g stock solution (b) was prepared by adding 3.28 g into 18 mL DI water and shaking until fully dispersed. 17 mL DI water containing 5 mg of MNPs was added to a plastic centrifugation tube, followed by adding 1 mL of (a), and placed into an ultrasonic ice bath for sonication. Next, 2 mL of solution (b) was rapidly added and hand‐shaken, after which sonication was continued for 1 h. The precipitate appeared after 3 h of steady state in the solution. A total volume of 20 mL of DI water was used. The particles were left undisturbed overnight, collected the next day by centrifugation, washed 3–4 times with DI water to remove residual precursors and byproducts, and then subjected to magnetic separation.

### 4.4 Synthesis of MZIF‐L Belts

A 2 m aqueous sodium hydroxide (NaOH) stock solution was prepared. The presence of NaOH modulates the deprotonation and coordination kinetics of 2‐MeIm, allowing for the particles to elongate along the [010] direction. The synthetic steps were the same as for “leaves”, except that at the step of addition of 2‐MeIm (solution (b)), it was first mixed with 0.1–0.3 mL of NaOH stock solution, and then rapidly added to a plastic tube containing the same number of precursors. A total volume of 20 mL DI was used for each reaction, and the pH values varied from 6 to 9, where “belts” were obtained at pH 9. When the pH was basic, the particles precipitated immediately upon addition of 2‐MeIm; therefore, hand‐shaking for 1 min after addition was recommended for homogeneous mixing. The precipitate was left in an ultrasonic ice bath for 1 h and then kept undisturbed overnight. The particles were washed thoroughly by centrifugation with DI water, replacing it 4–5 times until the final pH was neutral, and then separated by magnet, discarding the supernatant and re‐dispersing with DI water again. The MZIF‐L morphologies were reproducibly obtained across more than 10 independent synthesis batches prepared under the same conditions. Their aspect ratio (length/width) was calculated from image analysis of more than 30 particles per sample per batch. The same morphologies were also obtained after a tenfold scale‐up, indicating that the high‐aspect‐ratio morphology was not limited to a single synthesis batch.

### 4.5 Synthesis of ZIF‐L Leaves and Belts

The synthesis was the same as for magnetic counterparts, except that the MNPs were not added during the initial stages, while the number of precursors, the total volume of DI water, the washing and separation procedures remained the same.

### 4.6 Handling of Materials

Each synthesis with the specified number of precursors yields about 100–120 mg of dry powder, corresponding to an 85–100% near‐quantitative yield based on the Zn^2+^ precursor amount. The procedure was scaled up tenfold to produce gram‐scale quantities and ensure reproducibility. For analysis and characterization, the particles were centrifuged to remove water and dried in an open plastic container in a convection oven at 60°C overnight.

### 4.7 Characterization

SEM imaging was performed on a Hitachi S‐3400N, where the samples were drop‐casted onto carbon tape and sputtered with gold upon drying. The length and width of the MZIF‐L particles were measured by Image‐J analysis of the SEM micrographs with N = 30 measurements, then the data were transferred to OriginPro software to plot histograms with binomial regression curve, and Python via Matplotlib was used to calculate and add Poisson error bars. TEM was performed on an FEI Tecnai G2 F30, with the samples cast on a holey carbon substrate and dried overnight. XRD data were collected on a Rigaku Miniflex diffractometer from 2θ = 4° to 35°, using Cu Kα radiation (λ = 0.154 nm). The ZIF‐L crystal structure was rendered using CrystalMaker software from a crystallographic information file (CIF) reported in previous works on ZIF‐L [19, 24]. TGA was performed using a TA Instruments Q600 analyzer from 50°C to 630°C at a heating rate of 20°C/min under a dry air atmosphere. FTIR spectroscopy was performed using a Bruker Vertex 70 spectrometer equipped with an attenuated total reflectance (ATR) accessory, with samples dispersed in water individually drop‐cast onto the ATR crystal and dried under vacuum for 3 h.

## Conflicts of Interest

The authors declare no conflicts of interest.

## Supporting information




**Supporting File**: smtd70745‐sup‐0001‐SuppMat.pdf.

## Data Availability

The data that support the findings of this study are available on request from the corresponding author. The data are not publicly available due to privacy or ethical restrictions.
