# Supplementary material for: Strain‐Mediated Anisotropic Growth of Metal–Organic Framework Belts
Source: Small Methods. 2026 Jun 2;10(13):e70745. doi: 10.1002/smtd.70745 (PMC13353868; doi:10.1002/smtd.70745)
Supplement: Supplementary file 1 — Supporting File: smtd70745‐sup‐0001‐SuppMat.pdf. [file SMTD-10-e70745-s001.pdf]

Supporting Information

Strain-Mediated Anisotropic Growth of Metal Organic Framework Belts

Valeriia Poliukhova[1], Hsien Cheng Huang[1], Vladimir V. Tsukruk[1,*]

[1] School of Materials Science and Engineering, Georgia Institute of Technology, Atlanta, Georgia 30332, United States

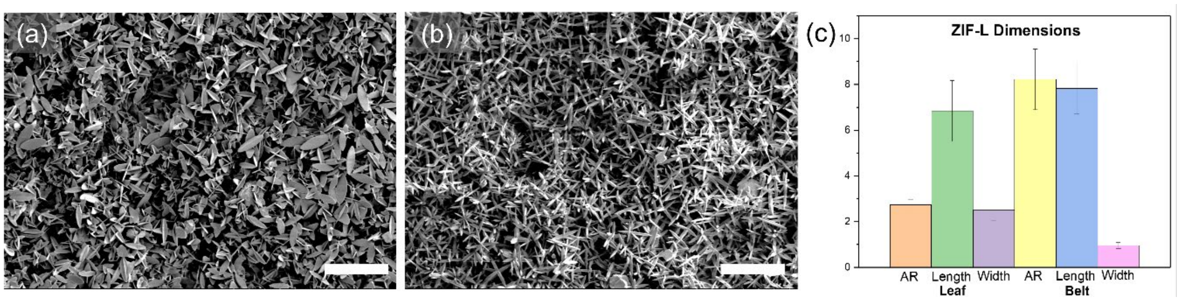

Figure S1. SEM images of obtained ZIF-L_leaf (a) and ZIF-L_belt (b) morphologies, and their dimensions, including aspect ratio (AR), length and width (c), synthesized without the addition of MNPs; scale bars 20 μm.

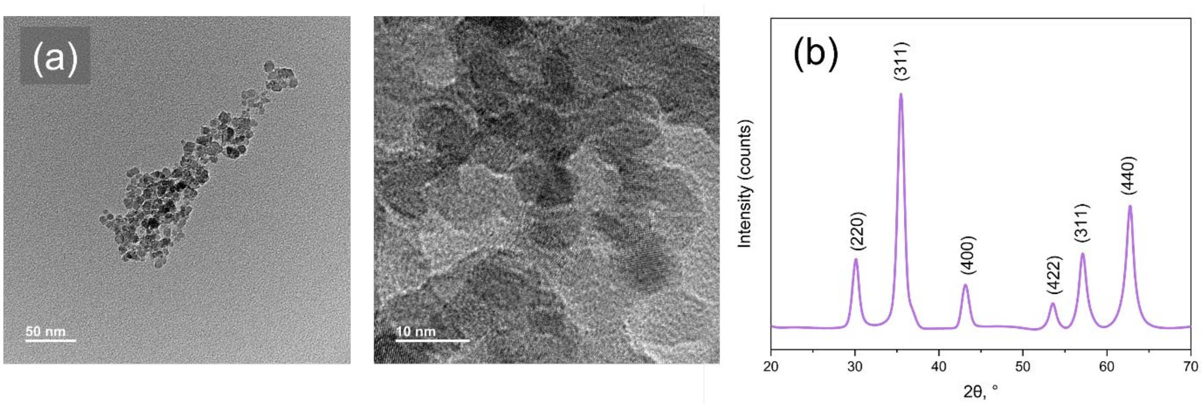

Figure S2. TEM images (a) and X-ray (b) of synthesized Fe_3O_4 magnetic nanoparticles (MNPs) with representative magnetite peaks, used in-situ during the ZIF-L formation to obtain MZIF-L in this study.

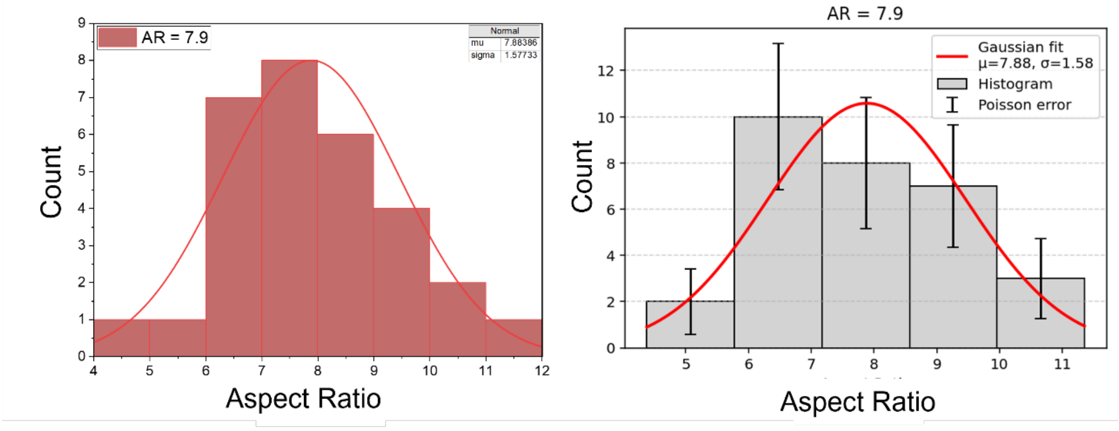

Figure S3. Representative histograms of the MZIF-L "belt" aspect ratio (AR) calculated from the measured SEM images and processed and plotted with Gaussian fit and Poisson error.

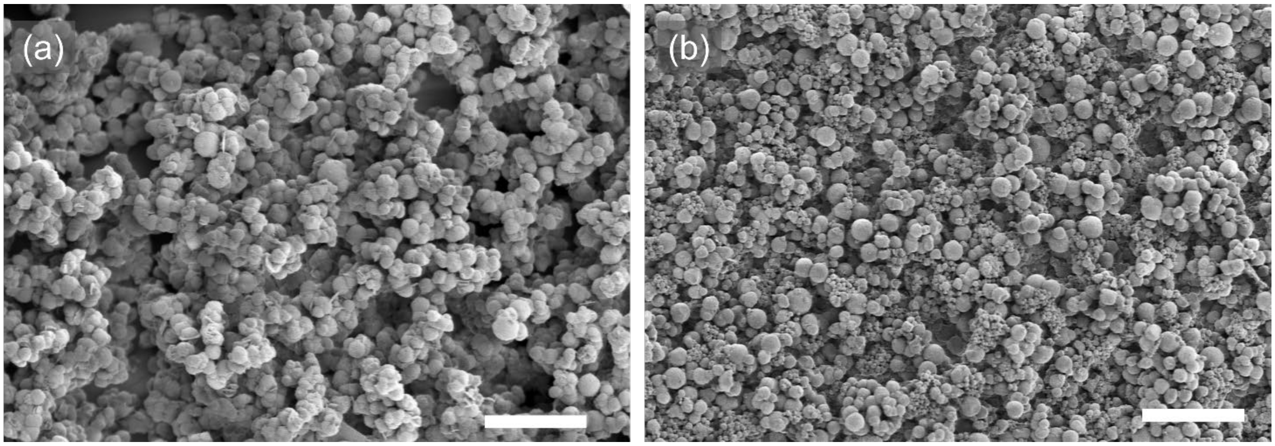

Figure S4. SEM images of the obtained MZIF-L spherical morphologies synthesized when the pH of the aqueous medium was 9.5 (a) and 10 (b), respectively. Scale bars 20 µm.

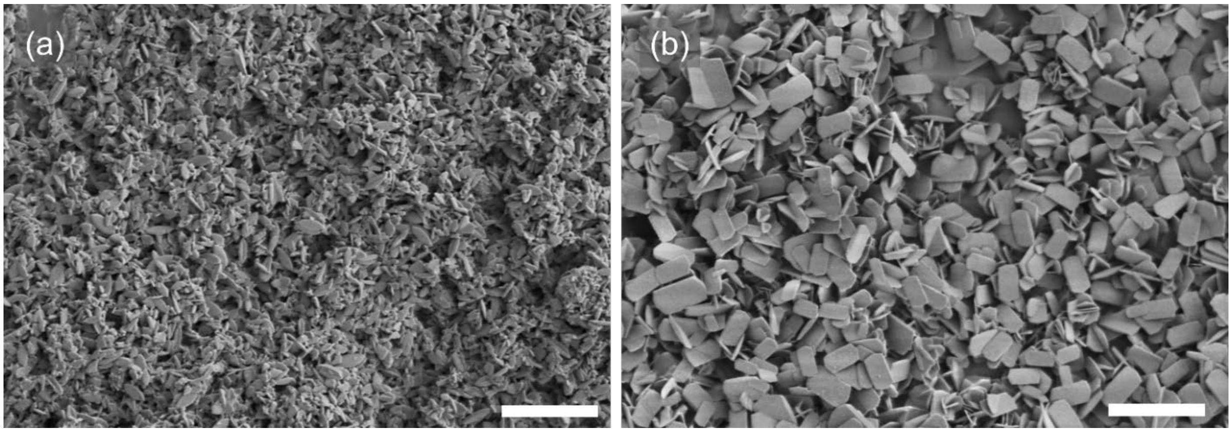

Figure S5. SEM images of the obtained MZIF-L morphologies synthesized with the pH = 6 and the total volume of water was 8 mL (a) and 100 mL (b), respectively. Scale bars 10 µm.

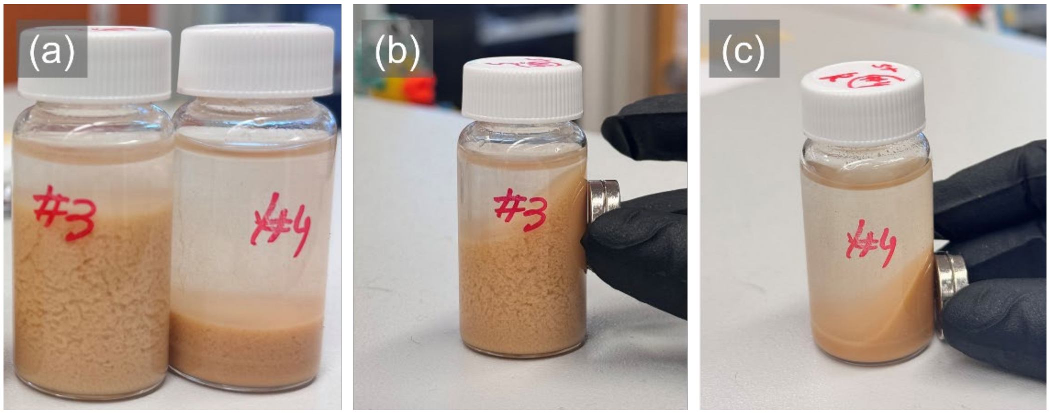

Figure S6. Photographs of freshly synthesized MZIF-L "leaf" and "belt" morphologies dispersed in DI water (a), and photographs showing how "leaf" (b) and "belt" (c) particles react when the 200 mT coin magnet is attached to a vial.

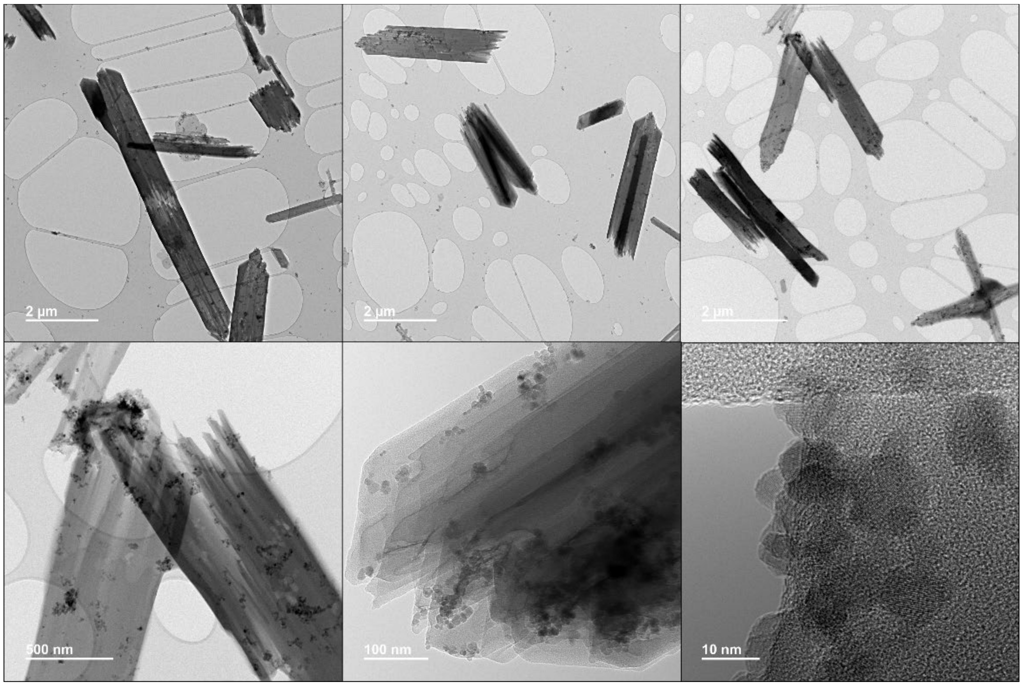

Figure S7. TEM of MZIF-L "belt" morphologies at different locations in low magnification (top panel, scale bar 2 µm), and higher magnification showing Fe_3O_4 MNPs attachment on the surfaces and layers of the belts.

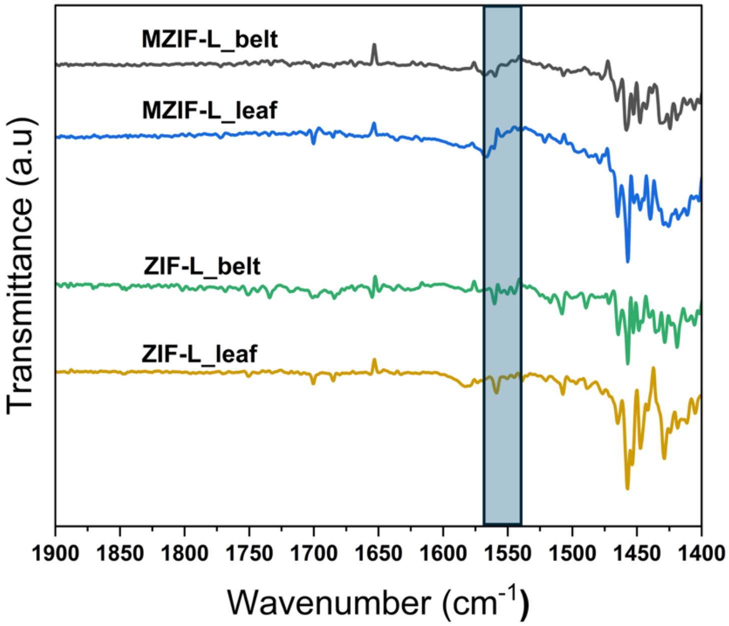

Figure S8. ATR-FTIR spectra of ZIF-L_leaf, ZIF-L_belt, MZIF-L_leaf, and MZIF-L_belt.

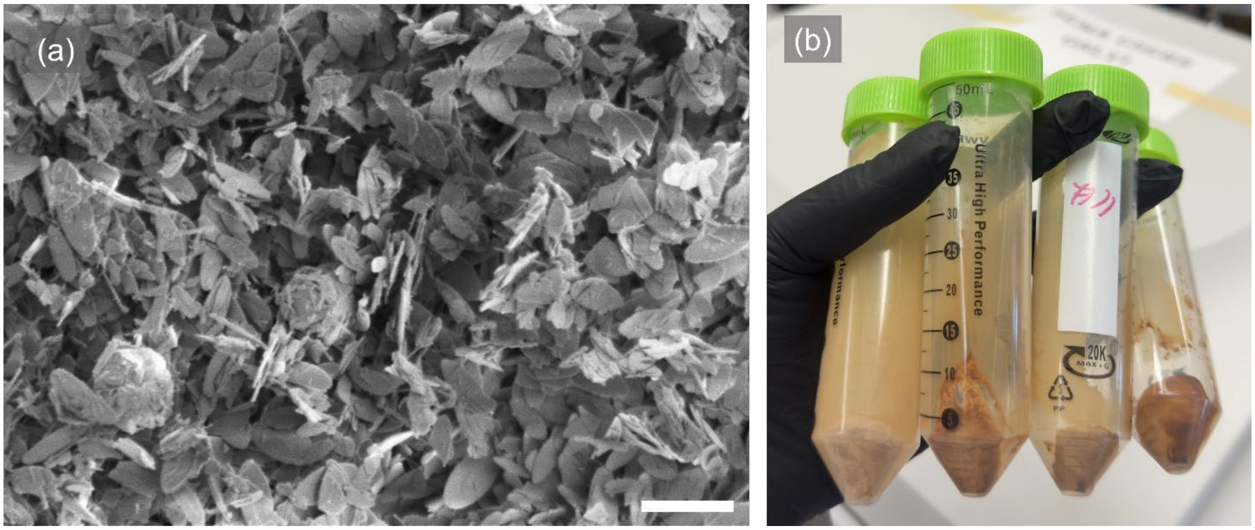

Figure S9. SEM micrograph (a) and photograph (b) of freshly synthesized MZIF-L "leaf" in the presence of Fe_3O_4 MNPs that did not have citric acid coatings on the MNPs. Photo shown after centrifugation. Scale bar 5 µm.

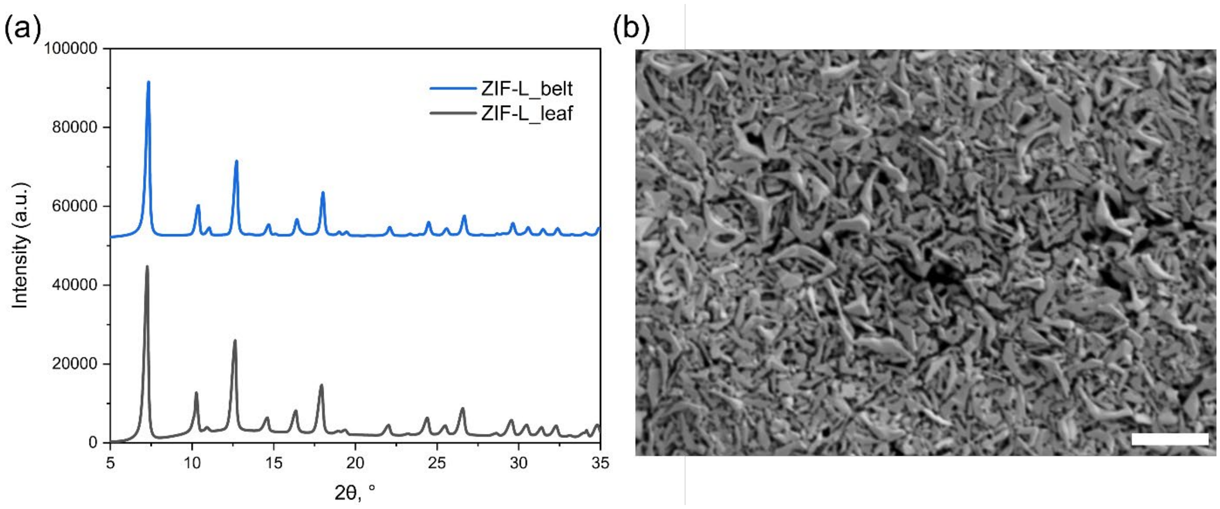

Figure S10. X-ray diffraction patterns of ZIF-L_leaf and ZIF_L belt powders that were dried for 24 hrs at 40°C, which show ZIF-8 fingerprint instead of ZIF-L (a). These patterns match the characteristic low-angle ZIF-8 reflections, with the strongest peaks at 2θ = 7.35°, 10.41°, 12.76°, 14.74°, 16.49°, and 18.08° (ICDD PDF 02-002-8846). An SEM image of ZIF-L_leaf powder after X-ray measurements; scale bar is 5 µm (b).

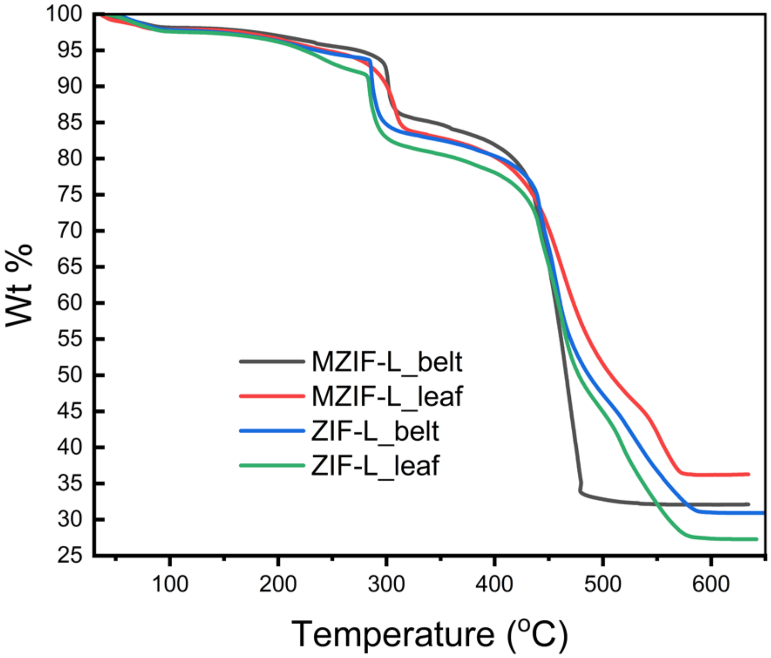

Figure S11. TGA of ZIF-L_leaf, ZIF-L_belt, MZIF-L_leaf, and MZIF-L_belt samples. All samples show multistep mass loss associated with adsorbed or interlayer water, weakly bound or free ligand species, and final framework degradation. The TGA curves provide thermal stability information, while XRD is the primary method for distinguishing ZIF-L phase retention from ZIF-8 reconstruction.
